# Precise delineation of radiation targets for thoracic tumors in the immunotherapy era: from immune mechanisms to clinical practice

**DOI:** 10.1002/pro6.70090

**Published:** 2026-08-30

**Authors:** Xiaoying Chen, Minxiao Yi, Yu Deng, Qian Shen

**Affiliations:** ^1^ Department of Oncology Tongji Hospital, Tongji Medical College, Huazhong University of Science and Technology Wuhan China; ^2^ Tongji Medical College, Huazhong University of Science and Technology Wuhan China; ^3^ Department of Thoracic Surgery Tongji Hospital, Tongji Medical College Huazhong University of Science and Technology Wuhan China

**Keywords:** Esophageal cancer, Lung cancer, Radiotherapy, Target delineation, Immunotherapy

## Abstract

As a core modality for local control of thoracic tumors, including lung and esophageal cancers, radiotherapy (RT) shows synergistic effects with immunotherapy, fundamentally reshaping the landscape of cancer treatment. Given the rapid advancements in immunotherapy, this review explores the critical role of precise delineation of radiotherapy targets in achieving synergistic effects between RT and immunotherapy. It elucidates the dual‐edged nature of RT, which can both activate antitumor immune responses and suppress immune function. Notably, larger radiation target volumes often cause destruction of tumor‐draining lymph nodes and reduce the number of peripheral blood lymphocytes, thereby impairing the efficacy of immunotherapy. To address this challenge, we discuss strategies tailored to thoracic tumors, including reducing target volumes, optimizing irradiation fields, and adjusting target positioning to protect key immune components. Furthermore, we highlight recent advancements in multimodal imaging, real‐time dynamic tracking technologies, and deep learning‐enabled automated segmentation methods that can increase the precision of target delineation. Additionally, emerging RT techniques, such as proton therapy and FLASH‐RT, demonstrate unique advantages in minimizing irradiation of normal tissues and circulating immune cells while enhancing immunomodulatory effects and thereby facilitating precise RT. This review aims to provide a theoretical foundation and technical support for accurate target delineation in the era of immunotherapy, particularly for thoracic malignancies, to promote the deep integration of RT and immunotherapy and improve patient outcomes.

## Graphical abstract

RT‐immunotherapy synergy for thoracic tumors relies on precise target delineation to protect immune components. IFI, reduced volumes, and optimized positioning minimize damage, while multimodal imaging, DL, PBT, and FLASH‐RT enhance control. These innovations shift RT to an immune‐centric model, improving lung and esophageal cancer outcomes.

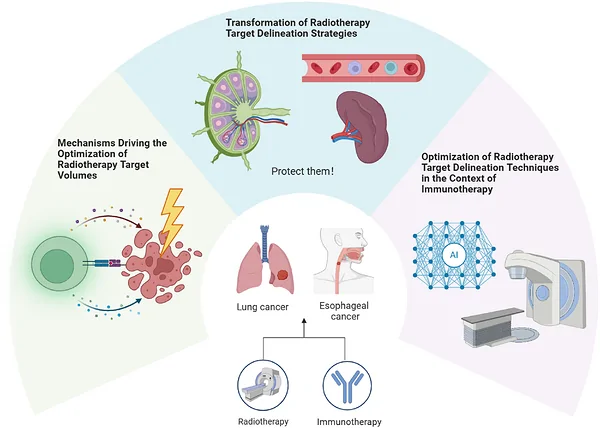

## 1 INTRODUCTION

With the ongoing advancements in cancer treatment, radiotherapy (RT) and immunotherapy have become pivotal for managing thoracic malignancies. The combination of RT and immunotherapy shows complementary biological advantages.^1^RT releases antigens by destroying tumor cells, activating the immune system and improving the tumor microenvironment (TME) while weakening immunosuppression; on the other hand, immunotherapy enhances systemic immune attacks and durable memory. Recent studies (Table 1, ^2^, ^3^, ^4^, ^5^, ^6^, ^7^, ^8^, ^9^, ^10^, ^11^) evaluating combinations of RT and immunotherapy for lung and esophageal cancer have highlighted the potential of this combined approach. Although the success of this combination hinges on immune system integrity, traditional target delineation strategies in RT may compromise this aspect. Conventional target delineation is primarily anatomy‐based, which frequently employs elective nodal irradiation (ENI) as a standard strategy. Although ENI reduces the risk of local recurrence, extensive irradiation also damages immune structures, thereby weakening immunotherapy efficacy. This issue is pronounced in thoracic tumors such as lung and esophageal cancer due to their proximity to the thymus, mediastinal, and axillary lymph nodes, where overirradiation can impair immunity and present the risk of complications such as pneumonia and esophagitis. Additionally, most of the clinical trials summarized in Table 1 do not explicitly report whether elective nodal regions are included in the irradiation field, highlighting the lack of standardized radiotherapy target‐volume strategies for combined RT and immunotherapy. Novel approaches such as involved‐field irradiation (IFI) target visible lesions, minimize immune damage, and optimize RT‐immunotherapy synergy. To achieve precise target delineation, the application of techniques such as positron emission tomography (PET)/computed tomography (CT), PET/magnetic resonance imaging (MRI), four‐dimensional (4D)‐CT, and image‐guided radiotherapy (IGRT) can integrate metabolic and anatomical data to pinpoint tumor boundaries and motion,^12^ thereby refining RT planning. Deep learning (DL)‐driven segmentation tools can reduce variability, identify lymph nodes, assess tumor‐infiltrating lymphocytes (TILs), and predict metastasis risk, facilitating planning for lung and esophageal cancer.^13^ Emerging RT methods such as proton therapy and FLASH‐RT use unique dose‐deposition methods to deliver high doses to tumors while sparing immune organs like the heart, lungs, and lymph nodes, creating ideal conditions for immunotherapy.

**TABLE 1:** Efficacy of Combined Radiotherapy and Immunotherapy in Patients with Lung and Esophageal Cancer.

Cancer	Study	Study Design	Patients	Classification	Enrollment	Treatment protocol	Result	Cite
Lung cancer	Willemijn et al., 2021	pooled analysis (PEMBRO‐RT and MDACC trials)	patients (aged ≥18 years) with metastatic NSCLC	Experimental	76	Pembrolizumab 200 mg Q3W + RT RT: 24 Gy/3 fx sequential [PEMBRO‐RT]; 50 Gy/4 fx or 45 Gy/15 fx concurrent [MDACC]	Best ARR:41.7% median PFS:9.0 months median OS:19.2 months	^1^
				Control	72	Pembrolizumab alone: 200 mg Q3W	Best ARR:19.7% median PFS:4.4 months median OS:8.7 months	
	Joe et al., 2023	randomised Phase II	people with early‐stage NSCLC	Experimental	66	SABR + nivolumab 480 mg Q4W ×4 (concurrent); SABR 50 Gy/4 fx or 70 Gy/10 fx with SIB	4‐year EFS:77%	^2^
				Control	75	SABR alone (50 Gy/4 fx or 70 Gy/10 fx)	4‐year EFS:53%	
	Shuling et al., 2022	retrospective study	patients with stage III and IV NSCLC	Experimental	119	Sintilimab Q3W + RT (CRT or SBRT to primary or metastatic sites)	The median PFS:9.00 months (95%CI 5.95‐12.05)	^3^
				Control	140	Sintilimab Q3W	The median PFS:5.00 months (95%CI 4.38‐5.62)	
	James et al., 2020	Phase I/II	patients who aged ≥18 years, with mNSCLC	Experimental	40	Pembrolizumab 200 mg Q3W + concurrent RT (50 Gy/4 fx SBRT or 45 Gy/15 fx)	median PFS:9.1 months (95% CI 3.6 to 18.4 months)	^4^
				Control	40	Pembrolizumab 200 mg Q3W	median PFS:5.1 months (95% CI 3.4 to 12.7 months)	
	Henrik et al., 2022	Phase II	patients with advanced NSCLC (stages III–IV) previously treated with a platinum doublet	—	21	Atezolizumab 1200 mg IV Q3W (≤2 years) + SBRT 6 Gy ×3 (administered between first and second infusion; at least one lesion left unirradiated)	PR:4 patients SD:8 patients PD:5 patients Median PFS:4.3 months (95% CI 2.2–8.7)	^5^
Esophageal cancer	Minghao et al., 2024	Phase Ib	patients with ESCC	—	18	41.4 Gy of radiation+ 4 cycles of 240 mg of toripalimab injection before R0 surgery	PCR:47.4% major PCR:68.4%	^6^
	Wencheng et al., 2021	Phase Ib	patients locally advanced ESCC	—	20	60 Gy radiation (2.0 Gy/fx, 5 fx/week)+ camrelizumab (200 mg every 2 weeks) starting with RT and continuing for 32 weeks (i.e., for 16 cycles)	24‐month OS:31.6% 24‐month PFS:35.5%	^7^
	Wensi Zhao et al., 2023	Phase II	oligometastatic ESCC patients after first‐line immunotherapy plus chemotherapy failure	—	49	IMRT (40 Gy/20 fx or 30 Gy/10 fx) + camrelizumab 200 mg Q3W + irinotecan 125 mg/m^2^ D1,8 Q3W	median PFS:6.9 months(95%CI, 4.6–9.3) median OS:12.8 months (95%CI, 10.1–15.5) ORR:40.8% (95%CI, 27.3–55.7). DCR:75.5% (95%CI, 60.8–86.2). ARR:34.7% (95%CI, 22.1–49.7). ACR:69.4% (95%CI, 54.4–81.3)	^8^
	Qi Zhao et al,. 2024	Phase Ib	patients advanced ESCC	—	25	KN046 (1–5 mg/kg) + cisplatin/paclitaxel Q3W + palliative RT (investigator discretion)	ORR:41.7% (95%CI 22.1‐63.4) DCR:87.5% (95%CI 67.6‐97.3) median PFS:7.8 months (95%CI 5.2‐9.7) median OS:15.9 months (95%CI 8.4‐NE)	^9^
	Tom van den Ende et al,. 2021	Phase II	patients with resectable esophageal adenocarcinoma (rEAC)	—	40	CROSS nCRT + atezolizumab 1200 mg (5 cycles)	PCR:25% (10/40) Baseline expression of an established IFNγ signature was higher in responders compared with nonresponders (P = 0.043)	^10^

Abbreviations: RT: radiotherapy, NSCLC: non‐small cell lung cancer, ARR: out‐of‐field (abscopal) response rate, PFS: progression‐free survival, OS: overallsurvival, SABR: Stereotactic Ablative Radiotherapy, SIB: Simultaneous Integrated Boost, EFS: event‐free survival, PR: partial response, SD: stable disease, PD: progressive disease, PCR: pathologic complete response, ESCC: esophageal squamous cell carcinoma, ACR: abscopal disease control rate, ORR: overall response rate, rEAC: resectable esophageal adenocarcinoma, nCRT:neoadjuvant chemoradiotherapy, IFNγ: interferon gamma

## 2 MECHANISMS DRIVING THE OPTIMIZATION OF RADIOTHERAPY TARGET VOLUMES

### 2.1 Mechanisms of immunomodulation by RT

**FIGURE 1:**
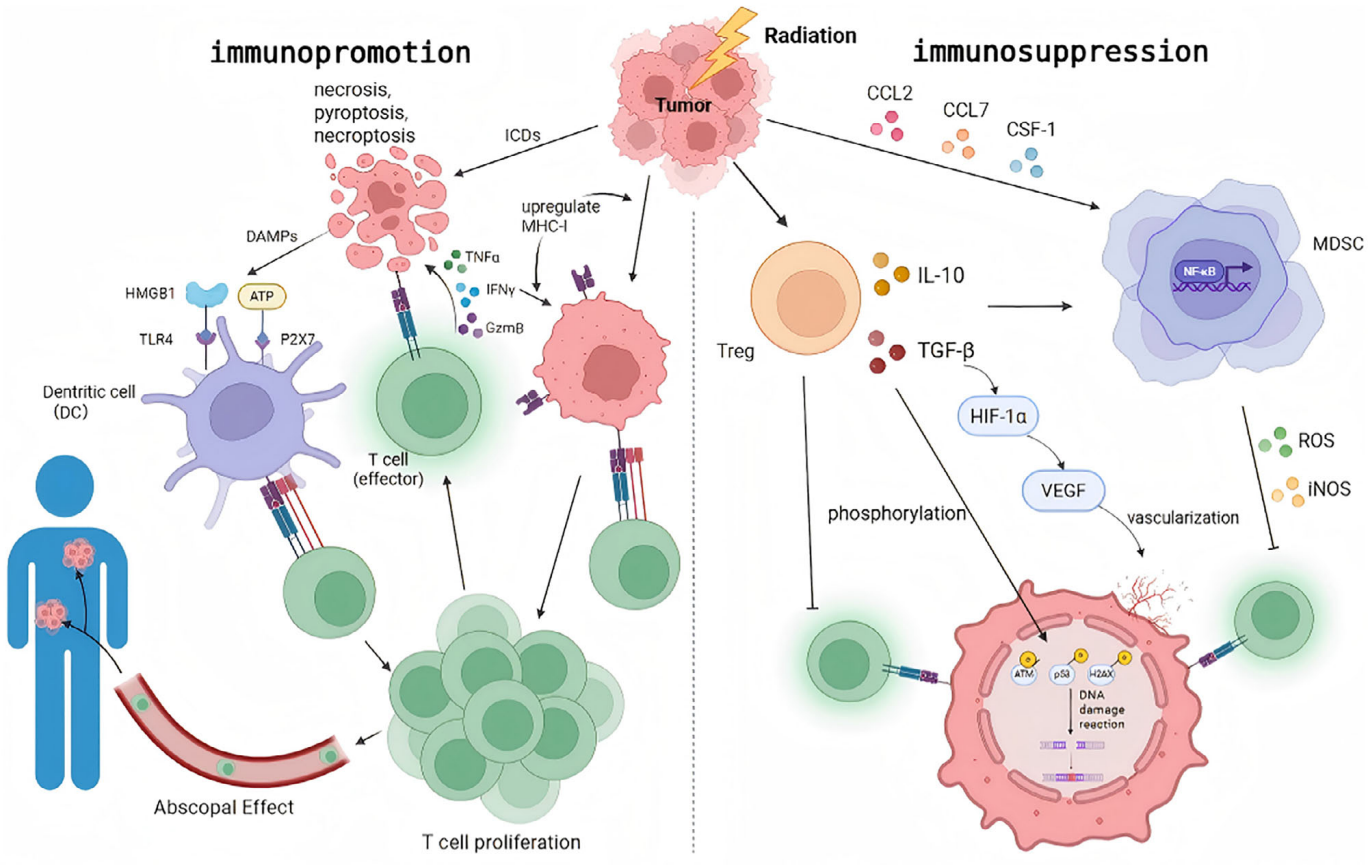
Radiation‐induced Immunomodulation in Tumors: The Balance between Immunopromotion and Immunosuppression. Created in https://BioRender.com.

RT can induce multiple forms of tumor cell death rather than a single uniform pattern, of which necrosis, necroptosis, and pyroptosis^14^, ^15^ are particularly associated with immunogenic cell death (ICD). These immunogenic death pathways release damage‐associated molecular patterns (DAMPs) and cytokines, creating an “in situ tumor vaccine” that activates systemic antitumor immune responses. This process involves tumor antigen release and presentation since RT‐induced ICD releases DAMPs to activate dendritic cells (DCs). DCs then present antigens to major histocompatibility complex class I (MHC‐I) molecules, activating endogenous CD8^+^ T cells.^16^ Ionizing radiation (IR) increases T cell infiltration and cytotoxicity by increasing the expression of tumor necrosis factor alpha (TNF‐α), interferon gamma (IFN‐γ), and granzyme B (GZMB).^17^, ^18^, ^19^ RT also upregulates MHC‐I molecules on the tumor cell surfaces to improve T cell recognition and killing.^20^ After immune signal amplification, radiation‐induced IFN‐γ further enhances MHC class I expression,^21^ and microparticles are released from irradiated tumor cells, increasing antigen presentation in nonirradiated cells through DNA repair pathways.^22^ These mechanisms underpin the synergy between RT and immunotherapy, which is critical for thoracic tumors where local immune activation improves prognosis.(Figure 1)

However, the immunomodulatory effects of RT have several limitations. Immunosuppression arises from the expansion of regulatory T cells (Tregs) and myeloid‐derived suppressor cells (MDSCs), where Tregs suppress effector T cells post‐RT by transforming growth factor‐β (TGF‐β) and interleukin (IL)‐10, also stimulating MDSC activity.^23^, ^24^, ^25^ RT promotes immunosuppression by recruiting MDSCs through the CCL2/CCL7/CSF‐1 axes and the cGAS‐STING/CCR2 pathway. Once in the TME, these MDSCs—augmented by RT‐induced hypoxia—suppress CD8^+^ T cell activity through the production of reactive oxygen species (ROS) and inducible nitric oxide synthase (iNOS).^26^ Additionally, MDSCs mediate “extrinsic radiation resistance” during RT by suppressing CD8^+^ T cell responses, which are linked to the activation of the nuclear factor (NF)‐κB signaling pathway.^27^ RT‐induced TGF‐β in the TME triggers the DNA damage response (DDR) in cancer cells through phosphorylation of H2AX, ATM, and p53, facilitating DNA repair, and promotes angiogenesis through hypoxia‐inducible factor‐1α and vascular endothelial growth factor, reducing cancer cell sensitivity to IR.^28^

RT can trigger the abscopal effect, wherein activated T cells circulate throughout the body, attacking distant tumor sites. However, this effect is rare with RT alone, with only 46 cases reported from 1969 to 2014.^29^ Combining RT with immunotherapy enhances abscopal response rates, overcoming immunosuppression. Localized RT with cytotoxic T‐lymphocyte‐associated protein 4 (CTLA‐4) blockade suppresses lung metastases in mice, whereas RT plus programmed death‐ligand 1 (PD‐L1) blockade increases T cell responses at primary and distant sites.^30^, ^31^, ^32^ The abscopal effect relies on immune system integrity, emphasizing the need to protect key immune components in thoracic tumor target volumes, since damage during RT impairs systemic immune responses.

### 2.2 Biological basis for the protection of key immune components

Tumor‐draining lymph nodes (TDLNs) are vital for antitumor immunity, driving systemic responses through antigen presentation and immune cell activation. Since TDLNs are the primary site where DCs activate antigen‐specific CD8^+^ T cells,^33^, ^34^, ^35^, ^36^ their integrity is key to the abscopal effect, especially in thoracic tumors near critical immune structures. Targeted irradiation of TDLNs, as shown in B16F10 models, reduces the number of tumor‐specific CD8^+^ T cells and long‐term immune memory mediated by stem‐like CD8^+^ T cells.^37^ The TDLN microenvironment supports CD8^+^ T cell survival and sustained antigen exposure,^38^ fueling systemic responses. Importantly, emerging clinical dose–volume evidence supports these findings. In patients with esophageal cancer treated with chemoradiotherapy, higher radiation exposure to radiologically negative TDLNs was associated with impaired peripheral T cell activation and significantly worse survival outcomes, indicating that excessive prophylactic irradiation of TDLNs may compromise antitumor immunity even in the absence of nodal metastasis.^39^ Therefore, preserving TDLNs is critical for RT and immunotherapy synergy. However, in clinical practice, TDLNs often harbor micrometastases, and sparing high‐risk nodal regions to preserve immunity may increase the risk of local recurrence.^40^ Thus, delineation of RT targets must carefully balance the preservation of immune function with adequate coverage of potentially involved lymph nodes. Consequently, protecting TDLNs is advantageous for synergizing RT with immunotherapy, provided that nodal coverage ensures sufficient local control.

Peripheral blood lymphocytes (PBLs) influence the efficacy of combined RT and immunotherapy. Lymphocytes are highly radiosensitive,^41^ with 0.5 Gy causing notable cell death and 3 Gy reducing the number of circulating lymphocytes by 90%.^42^ This makes them key indicators of RT‐induced immune damage. In locally advanced esophageal squamous cell carcinoma (LAESCC), a large gross tumor volume (GTV) is correlated with lymphocyte depletion and reduced overall survival (OS).^43^ Thus, precise RT target delineation must balance tumor eradication with protection of lymphocyte‐rich microenvironments to maintain immune function and control of tumors.

**FIGURE 2:**
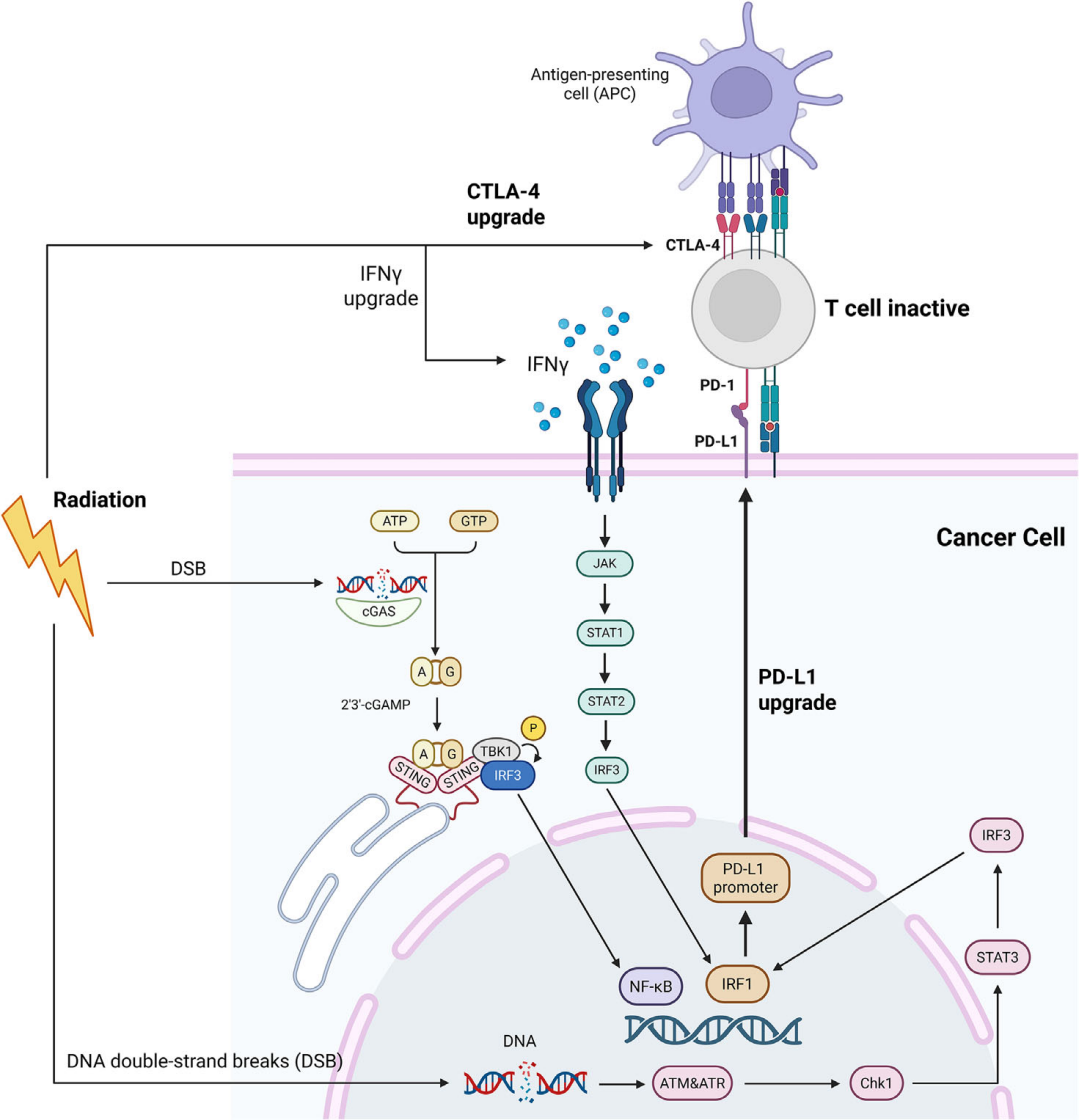
Radiation‐induced Signalling Pathways in Cancer Cell Immune Evasion. Created in https://BioRender.com.

In lung and esophageal cancer treatment, immunotherapy mainly involves immune checkpoint inhibitors (ICIs), such as inhibitors of coinhibitory molecules such as programmed death protein 1 (PD‐1)/PD‐L1 and cytotoxic T‐lymphocyte‐associated protein (CTLA‐4), which synergize with RT (Figure 2). RT upregulates PD‐L1 in tumors and their microenvironment via DNA damage/ATM/STAT3/IRF3, IFN‐γ/JAK/STAT/PD‐L1, and cGAS‐STING signaling. This upregulation weakens the effects of T cells.^44^, ^45^ However, PD‐L1 inhibitors block PD‐1/PD‐L1 interactions, relieving T cell suppression and restoring tumor‐killing capacity.^46^ As for CTLA‐4, when combined with RT, CTLA‐4 inhibitors can convert tumors unresponsive to anti‐CTLA‐4 therapy into responsive ones.^47^, ^48^ Since combined RT and immunotherapy efficacy relies on immune components, protecting TDLNs and lymphocytes during RT target delineation enhances effector lymphocyte function activated by ICIs, increasing treatment success.

## 3 TRANSFORMATION OF STRATEGIES FOR RT TARGET DELINEATION

### 3.1 Clinical practice and immunological rationale for the transition from ENI to IFI

The traditional strategy for ENI in RT targets the primary tumor and potential subclinical spread, including regional lymph nodes at risk of micrometastases, to reduce local recurrence rates. Although ENI improves local control, it damages the immune system, especially in ECs, where target volumes cover cervical and thoracic vascular and lymphatic tissues. Although it is based on the theoretical basis of prophylactic lymph node dissection,^49^ its negative influence on the immune system is significant.^50^ Clinical data also revealed that ENI does not improve long‐term survival in patients with EC and increases esophagitis and cardiopulmonary toxicity.^51^ This contradiction has driven a shift toward precision RT, transitioning from ENI to IFI.

IFI targets only imaging‐visible lesions and adjacent subclinical areas, excluding regional lymph nodes, to preserve the integrity of the lymphatic system while maintaining efficacy. Studies have shown that IFI achieves comparable survival outcomes for esophageal and lung cancer patients in comparison with ENI (Table 2, ^52^, ^53^, ^54^, ^55^, ^56^, ^57^, ^58^). The immunological rationale for IFI stems from the cancer‐immunity cycle.^59^, ^60^ Disruption at any step risks immune escape, emphasizing the integrity of the lymphatic system for combined RT and immunotherapy. In patients with inoperable stage III locally advanced non‐small cell lung cancer (NSCLC) receiving concurrent chemoradiotherapy, the IFI group showed better 5‐year OS and local control rates (LCRs) than the ENI group.^61^ For patients with EC, IFI matched ENI in terms of OS and LCR in addition to showing reduced toxicity, notably lower rates of grade ≥2 and grade ≥3 acute esophagitis.^62^, ^63^ These findings suggest that IFI represents a paradigm shift in RT during the immunotherapy era, moving from broad coverage to precise immune protection and laying the foundation for combined therapies.

**TABLE 2:** Comparative Analysis of ENI and IFI in Enhancing the Efficacy of Radiotherapy for Esophageal and Lung Cancer Patients.

Cancer	Study	Study design	Patient population	Treatment protocol	Survival Outcomes	Adverse Effects	Cite
lung cancer	Fernandes et al., 2010	Retrospective cohort study	Stage III/IV NSCLC, n = 108	ENI/IFRT, radiotherapy dose not specified, 95/108 received chemotherapy	2‐year OS: 43.7% (IFRT) vs. 40.1% (ENI)	High‐grade esophagitis: odds ratio 0.31 (IFRT), *P* = 0.036	^45^
	Fernandes et al., 2009	Retrospective cohort study	Stage III/IV NSCLC, n = 104	ENI: 6345 cGy; IFRT: 6988 cGy; chemotherapy not specified	No mention found	Decreased esophagitis with IFRT; no rates	^46^
	Topkan et al., 2020	Retrospective propensity score‐matched cohort study	Stage IIIB/C NSCLC, n = 1048 (propensity score‐matched: 323 ENI, 323 IFRT)	IFRT/ENI: 66 Gy, 2 Gy per fraction, daily; 1–3 cycles platinum doublet chemotherapy	Median OS: 24.6 months (IFRT) vs.25.2 months (ENI), *P* = 0.69; 5‐year OS: 20.8%; 10‐year OS: 11.5%	Grade 3–4: leukopenia 9.9% (IFRT) vs. 15.7% (ENI), esophagitis 9.0% vs. 15.2%, pneumonitis 8.4% vs. 14.5%	^47^
esophageal cancer	Wang et al., 2022	Systematic review and meta‐analysis (23 studies, n = 4120)	ESCC and esophageal adenocarcinoma (EAC), mostly late‐stage	3D CRT/IMRT, some with concurrent chemotherapy, IFI vs ENI	5‐year OS: IFI improved (risk ratio 0.78, 95% confidence interval 0.68–0.90, *P* = 0.0004)	IFI reduced grade 2/3 acute esophagitis (risk ratio 0.79/0.51, *P*<0.001); no difference in pneumonia	^48^
	Bai et al., 2016	Meta‐analysis (12 trials, n = 1095)	Esophageal cancer	3D CRT/IMRT, IFI vs ENI	1‐, 3‐, 5‐year OS: odds ratios 0.966, 0.946, 0.732 (all not significant)	ENI higher grade 3 pneumonitis (odds ratio 0.348, *P*= 0.001), esophagitis (odds ratio 0.385, *P*= 0.000)	^49^
	Cheng et al., 2018	Meta‐analysis (10 studies, n = 1348)	ESCC, locally advanced	3D CRT/IMRT, some with chemotherapy, IFI vs ENI	No significant difference at 1‐, 2‐, 3‐year OS	IFI lower grade 3 esophagitis (odds ratio 0.467, *P* = 0.000), pneumonia (odds ratio 0.464, *P* = 0.008)	^50^
	Yamashita et al.,2015	Retrospective with prospective IFRT arm	239 patients, esophageal cancer, 24.7% stage IV	3D‐CRT, 50‐50.4 Gy, 1.8‐2 Gy/fraction, concurrent chemotherapy, IFRT vs ENI	1‐, 2‐, 3‐year OS: ENI 65.8%, 45.8%, 34.8%; IFRT 70.8%, 58.7%, 51.6%	ENI: leukopenia 49% (grade 3), 24% (grade 4); anemia 18% (grade 3), 12% (grade 4); thrombocytopenia 19% (grade 3), 17% (grade 4); esophagitis 23% (grade 3), 2% (grade 4); IFRT: lower rates	^51^

Abbreviations: ENI: elective nodal irradiation, IFRT: involved‐field radiotherapy, NSCLC: non‐small‐cell lung cancer, OS: overall survival, cGy: centiGray, ESCC: esophageal squamous cell carcinoma, EAC: esophageal adenocarcinoma, 3D CRT: three‐dimensional conformal radiotherapy, IMRT: intensity‐modulated radiotherapy, IFI: involved‐field irradiation

### 3.2 Immune protection strategies based on target‐volume reduction and positional optimization

Radiation‐induced lymphopenia (RIL) is closely related to the radiation target volume and significantly affects thoracic tumor RT prognosis and immunotherapy efficacy. Studies have shown that larger target volumes cause more severe RILs,^64^, ^65^ but a retrospective study on patients with NSCLC revealed that patients with lymphopenia had shorter progression‐free survival (PFS) and OS than those without lymphopenia.^66^ Moreover, recent clinical evidence directly linked RIL to impaired immunotherapy outcomes: in a cohort of 167 patients with esophageal squamous cell carcinoma receiving PD‐1 inhibitors, treatment‐related lymphopenia was an independent prognostic factor for poorer PFS, and in patients with advanced NSCLC, lymphopenia was associated with worse prognosis and correlated with more intensive radiotherapy.^67^, ^68^ Therefore, reducing the target volume can mitigate RILs and increase tumor responsiveness to immunotherapy.

Clinical studies on locally advanced (LA)‐NSCLC have demonstrated that reducing or omitting the clinical target volume (CTV) is safe and reduces radiotherapy‐related toxicity. In a phase II clinical trial by Cui et al., 90 patients with inoperable stage III NSCLC underwent PET/CT‐guided CTV omission and showed lower rates of severe radiation‐related pulmonary events and grade 3+ esophagitis due to reduced irradiation of the lungs and esophagus.^69^ Zou et al. studied 63 patients who underwent IMRT with chemotherapy and reported that CTV omission did not increase subclinical failure and may reduce lymphopenia.^65^ This immune‐protective effect may create favorable conditions for subsequent immunotherapy.

The anatomical location of the target volume also influences lymphocyte damage. RT to the spine, mediastinum, and chest wall more readily decrease the peripheral absolute lymphocyte count (ALC) than brain or limb irradiation do, likely due to greater exposure of the blood and bone marrow.^70^ Therefore, organs‐at‐risk (OAR) in RT are being increasingly conceptualized as immune‐related OARs (Immune‐OARs), which encompass highly perfused organs (like the heart) and lymphoid‐rich hematopoietic organs (like the spleen).^51^ To quantitatively assess the cumulative impact on the immune system, the effective dose to immune cells (EDIC) model has been proposed as a surrogate marker for RIL and prognosis in thoracic malignancies. The EDIC model integrates mean doses to highly perfused organs, including the heart, lungs, and major blood pools, to estimate the cumulative radiation exposure to circulating immune cells.^71^, ^72^ In other tumor types, higher radiation doses to the cervical spine, jugular vein, and spleen increase RIL risk,^73^, ^74^ a pattern also observed in thoracic tumors, and spleen dosimetry in gastroesophageal cancer treatment correlates with lymphocyte loss.^75^ Thus, RT‐induced immune damage is related to the immune‐OAR dose, and minimizing irradiation of key immune organs in RT planning can reduce RIL, enhancing the immunotherapy response.

## 4 OPTIMIZATION OF TARGET DELINEATION TECHNIQUES FOR RT IN THE CONTEXT OF IMMUNOTHERAPY

### 4.1 Multimodal imaging‐assisted target delineation: precise localization of tumors and immune metabolic activation zones

#### 4.1.1 Synergistic mechanisms of PET and CT/MRI

In imaging diagnosis and RT target delineation for thoracic tumors, multimodal imaging approaches, such as PET/CT and PET/MRI, combine the strengths of individual imaging modalities. PET/CT merges the functional insights from PET with the anatomical detail derived from CT, improving tumor localization and staging.^76^ In NSCLC, the GTV defined by PET/CT is smaller than that defined by CT alone, improving target coverage and reducing doses to organs at risk.^77^ This precision boosts tumor control, reduces normal tissue complications, and improves outcomes.^78^ PET/MRI combines metabolic data from PET with the anatomical and physiological insights from MRI.^79^, ^80^ While the GTV is typically based on the tumor morphology on MRI, the biological data from PET can reduce marginal misses attributable to inaccurate boundary assessment, facilitating GTV adjustment.^81^ Approximately 90% of GTV‐PET images overlap with GTV‐MRI images, but 10% of tumor and lymph node volumes are uniquely identified by PET, underscoring the value of ^18^F‐FDG PET/MRI in RT planning.^82^ Integrating PET and MRI data allows precise tumor boundary definition, minimizes irradiation to critical organs such as the spinal cord and lungs, reduces RT side effects, and enhances treatment precision and personalization.^83^ Critically, by distinguishing viable tumors from atelectasis and identifying CT‐occult nodal disease, multimodal imaging provides a robust radiological basis for clinical decision‐making.^84^, ^85^ This precision facilitates the transition from ENI to IFI, allowing selective nodal coverage without compromising regional control.

#### 4.1.2 Real‐time dynamic tracking of tumor displacement

During RT, thoracic tumors and the surrounding organs often shift due to respiration and heartbeat, causing the target volume to deviate from its planned position. Real‐time tracking technologies such as IGRT and 4D‐correlated dynamic tracking address this challenge. IGRT involves real‐time monitoring of tumor position by the integration of imaging techniques (such as CT) with RT equipment, allowing corrections to the position and dose distribution and reducing targeting errors.^86^ IGRT also analyses movement trends, guiding planning target volume (PTV) expansion for safer, precise stereotactic body radiotherapy (SBRT).^87^ The respiratory motion monitoring in IGRT allows comparison of planned motion amplitudes with the findings of daily assessments,^88^, ^89^, ^90^ which is crucial for the treatment of lung cancer. 4D‐CT dynamically tracks tumor displacement by adding a temporal dimension to the data obtained from three‐dimensional (3D)‐CT imaging, capturing organ and tumor changes across the respiratory cycle. This approach, which is widely used for lung and esophageal cancers,^91^ accurately measures lung tumor positions and predicts motion patterns, improving tracking efficiency during RT. It can also track tumors near the diaphragm and extrapulmonary tissues affected by respiration.^92^ In combination with fiducial markers, 4D‐CT can reduce motion‐induced blurring in 4D cone‐beam CT (CBCT) and digital tomosynthesis^93^ and allow monitoring of mediastinal lymph node motion, facilitating precise target‐volume definition. Beyond improving real‐time localization, motion‐managed imaging can directly influence target‐volume delineation and planning decisions. Four‐dimensional CT allows individualized definition of the internal target volume (ITV) by incorporating tumor motion across the respiratory cycle, thereby reducing geometric uncertainty in GTV delineation in comparison with conventional CT alone.^94^ Respiration‐correlated image guidance, including 4D‐CBCT, can further improve localization accuracy and reduce inter‐observer variability, supporting tighter PTV margins and more consistent target definition in lung RT, particularly for SBRT.^95^

### 4.2 Deep learning (DL) empowers precision therapy

DL technologies, particularly convolutional neural networks (CNNs), have emerged as a focus for automated image segmentation.^96^ In thoracic RT, the use of DL technologies can increase contouring accuracy and consistency, reduce human error, and decrease planning time. For example, Bi et al. reported that DL‐assisted contouring (DLAC) improved the accuracy, consistency, and efficiency of CTV in patients with NSCLC receiving postoperative radiotherapy (PORT).^97^ Clinical evaluations have indicated that DL‐generated contours accelerate OAR delineation for lung cancer.^98^ Moreover, the 4D‐CT‐based DL framework proposed by Yang et al. enables automatic delineation of the internal GTV, leveraging higher contrast and clearer boundaries for precise contouring, potentially improving prognosis.^99^

Beyond anatomical structures, DL can also facilitate the identification of immune‐related features within the TME, such as TILs, which play a critical role in the TME and are closely related to immunotherapy response and patient prognosis. Manual TIL evaluation lacks reproducibility, making accurate detection vital. The hybrid DL model proposed by Ebrahimi et al. predicts lymphocyte depletion trends in ECs, with a mean squared error (MSE) of 30% in comparison with traditional methods.^100^ In lung cancer, a machine learning approach can match existing standards for TIL quantification, facilitating digital pathology applications.^101^ DL can also refine lymph node delineation and metastasis prediction. The modified ResNet‐50 for endoscopic ultrasound (EUS) in EC proposed by Joseph et al. could distinguish malignant nodes from benign nodes, achieving 70% accuracy in subsets and 60% accuracy in larger tests.^102^ In predicting lymph node metastasis in thoracic tumors, Zhou et al. combined ultrasound images with DL models to allow early prediction of the metastatic risk of clinically negative axillary lymph nodes, providing more refined clinical treatment strategies.^103^

### 4.3 Immunological advantages of emerging radiotherapy technologies: balancing precision and efficiency

#### 4.3.1 Proton beam therapy

Proton beam therapy (PBT) is an advanced RT technique wherein high‐energy proton beams are used to target cancer cells while sparing healthy tissues. In comparison with photon‐based radiotherapy, PBT offers distinct dosimetric characteristics. PBT provides a precise dose distribution via the Bragg peak, where protons release most of their energy at a predetermined depth, dropping off sharply afterward. This allows the administration of high doses to tumors while minimizing harm to tissues beyond them,^104^ unlike photon RT, which irradiates healthy tissues to cause more side effects. A large retrospective analysis of 477 EC patients who were undergoing IMRT versus 250 who were receiving PBT revealed that PBT cut the mean heart dose and radiation to cardiac structures.^105^ Sejpal and colleagues reported that PBT reduced grade ≥3 pneumonitis and esophagitis rates (2% and 5%) in comparison with 3D conformal RT (30% and 18%) and IMRT (9% and 44%) in 62 stage III NSCLC patients.^106^ Notably, however, PBT does not automatically reduce geometric target volumes. Due to distal range uncertainty and sensitivity to respiratory motion, ITVs and PTV margins in PBT often remain comparable to those in photon therapy to ensure robust coverage.

The true advantage of PBT in the immunotherapy era lies not in aggressive margin reduction, but in its ability to protect the systemic immune system through its steep dose fall‐off. By minimizing the “low‐dose bath” to immune organs^107^ and circulating lymphocytes,^108^ PBT can significantly reduce the risk of treatment‐related immunosuppression. A retrospective analysis of 448 patients with EC receiving chemoradiotherapy revealed that PBT reduced the risk of severe lymphopenia in comparison with IMRT, especially in patients with lower esophageal tumors.^109^ In 223 patients with NSCLC, pencil beam scanning (n = 29) reduced the incidence of severe lymphopenia in comparison with IMRT (n = 194) by reducing the degree of lung irradiation.^110^ Beyond preserving lymphocyte counts, PBT further enhances immunomodulation at the cellular level. Gameiro et al. reported that^111^ surviving tumor cells after proton or photon radiation show immunogenic changes, such as the upregulation of surface molecules (human leukocyte antigen [HLA], intercellular adhesion molecule‐1 [ICAM‐1], and tumor antigens) and calreticulin, increasing the degree of killing by cytotoxic T lymphocytes (CTLs). This property also applies to cancer stem cells, where proton RT induces calreticulin upregulation, increasing their sensitivity to CTLs. In addition to enhancing T cell‐mediated immunity, proton therapy promotes macrophage polarization toward the M1 phenotype, increases the cytotoxicity of natural killer (NK) cells, and alters the immunosuppressive, recruitment, and differentiation properties of MDSCs.^112^ These immunomodulatory effects of PBT offer new strategies to improve treatment outcomes for patients with thoracic tumors.^113^, ^114^

Preclinical studies have shown the antitumor effects of 10–15 Gy in combination with PBT and immunotherapy in head and neck cancer models. However, clinical evidence for the efficacy of combining PBT with immunotherapy remains limited. Ongoing trials (e.g., NCT03765190, NCT03818776, NCT03267836, NCT03539198, NCT03764787, NCT06133062, and NCT05625893) have explored PBT and immunotherapy for various cancers, including NSCLC (Table 3). Further research is needed to clarify the underlying mechanisms and optimize PBT for thoracic tumors.^112^, ^115^

**TABLE 3:** Clinical Trial of Proton Radiotherapy Combined with Immunotherapy.

NCT number	Study title	Conditions	Radiotherapy	Immunotherapy	Primary Outcome Measures	Secondary Outcome Measures	Status	Enrollment	Date
NCT03765190	Combination of Proton Therapy With Immunotherapy in Multiple Metastases Cancer (PI‐MMC)	MMC	Proton Therapy	PD‐1 antibody	Adverse events	PFS, OS	Not yet recruiting	30	2018/12/3–2022/12/1
NCT03818776	Proton Based Cardiac Sparing Accelerated Fractionated RadioTherapy in Unresectable NSCLC	NSCLC	Proton beam therapy	Durvalumab	number of participants with DLT between first dose of Durvalumab and 30 days following completion of radiotherapy.	number of participants receiving full course of RT treatment and minimum of two doses of Durvalumab, number of participants with adverse events	Completed status (Results not yet available)	7	2019/8/30–2022/1/27
NCT03267836	Neoadjuvant avelumab and hypofractionated proton radiation therapy followed by surgery for recurrent radiation‐refractory meningioma	Meningioma	Proton radiotherapy	Avelumab	changes of CD8+/CD4+ TILs in recurrent radiation‐refractory meningioma	The number and percentage of subjects experiencing each type of adverse event, Radiological response, Pathologic response. PFS, OS	Completed status (Results not yet available)	9	2018/1/10–2023/4/6
NCT03539198	Study of proton SBRT and immunotherapy for recurrent/ progressive locoregional or metastatic head and neck cancer	Head and neck cancer	Proton SBRT	Nivolumab	ORR in locoregional and metastatic arm	LCR, Overall Survival Time, OS, PFS, TTP, new development of distant metastasis, quality of life, adverse effects, predictive and prognostic biomarkers for both arms	Completed status (Results not yet available)	19	2018/7/3–2020/1/6
NCT03764787	Combination of Hypofractionated Proton Therapy With Immunotherapy (I‐HypoPT)	Adult Solid Tumor	Hypofractionated Proton Therapy	PD‐1 antibody	Adverse events	PFS, OS	Not yet recruiting	30	2019/01/01–2021/12/01
NCT06133062	Atezolizumab and bevacizumab with proton radiotherapy for unresectable hepatocellular carcinoma	Hepatocellular carcinoma	Proton RT	Atezolizumab Bevacizumab	PFS	LC, TTP, ORR, OS, Incidence and severity of adverse events	Recruiting status	45	2023/11/16–2030/9/30
NCT05625893	Proton beam radiotherapy followed by tecentriq and avastin for primary liver cancer with Vp2‐4 portal vein invasion	Hepatocellular carcinoma	Proton beam therapy	Atezolizumab Bevacizumab	PFS, Incidence of adverse event	OS, TTP, ORR, DCR, LTP	Recruiting status	63	2022/9/16–2025/12/31

Abbreviations: MMC: Multiple Metastases Cancer, PD‐1: programmed death protein 1, PFS: progression‐free survival, OS: overall survival, NSCLC: non‐small cell lung cancer, DLT: dose limiting toxicities, RT: radiotherapy, TILs: tumor infiltrating lymphocytes, SBRT: stereotactic body radiation therapy, ORR: Objective response rate, LCR: local control rate, TTP: time to progression, LC: local control, DCR: disease control rate, LTP: local disease progression

#### 4.3.2 FLASH radiotherapy

In comparison with proton therapy, FLASH‐RT, which uses ultra‐high dose rates (≥40 Gy/s) delivered in a single fraction, balances immune protection and activation to offer unique biological effects. It maintains tumor control while reducing normal tissue toxicity, showing promise for thoracic tumors.^116^, ^117^, ^118^ FLASH‐RT has been shown to match or exceed conventional radiotherapy (CONV‐RT) in tumor suppression across models.^119^, ^120^, ^121^, ^122^, ^123^ It has been also shown to protect normal tissues, such as the lungs, skin, and intestines,^119^, ^124^, ^125^ reducing side effects and improving quality of life. In a lung cancer animal model, Favaudon et al.^119^ found that FLASH‐RT suppressed tumor growth in a manner similar to CONV‐RT but shielded lung tissue from fibrosis linked to TGF‐β and protected vascular, bronchial, and epithelial cells from apoptosis. Importantly, while FLASH‐RT spares normal tissues, it does not inherently reduce CTV. The ultra‐high dose rate influences dose distribution and may allow robust target coverage with reduced low‐ and intermediate‐dose exposure to surrounding organs, but ITVs and PTVs still need to be carefully defined to account for respiratory motion and setup uncertainties.

In addition to protecting normal tissues, FLASH‐RT offers notable advantages in immune preservation and is vital for combination with immunotherapy. It reduces circulating immune cell killing, with mouse models showing a decrease from 90%–100% with CONV‐RT to 5%–10% with FLASH‐RT at 40 Gy/s, especially at higher doses.^126^ Owing to this dose‐sparing effect on surrounding tissues, FLASH‐RT may also indirectly limit irradiation of circulating lymphocytes and immune‐related structures, contributing to immune preservation. Notably, FLASH‐RT has also shown long‐term advantages in immune system recovery. Tao et al.^127^ reported that FLASH‐RT preserved PBLs and increased bone marrow stem/progenitor cell proliferation and recovery in thoracic tumors. Furthermore, FLASH‐RT can activate the tumor immune microenvironment (TIME). Kim et al. reported that the MLC signaling pathway is upregulated in a Lewis lung cancer model,^128^ whereas other studies reported increased infiltration of macrophages, effector T cells, and other immune cells, turning “cold” tumors into “hot” tumors.^120^, ^129^, ^130^ These characteristics suggest that in combination with ICI drugs, FLASH‐RT may enhance immune recognition and antitumor effects.

**FIGURE 3:**
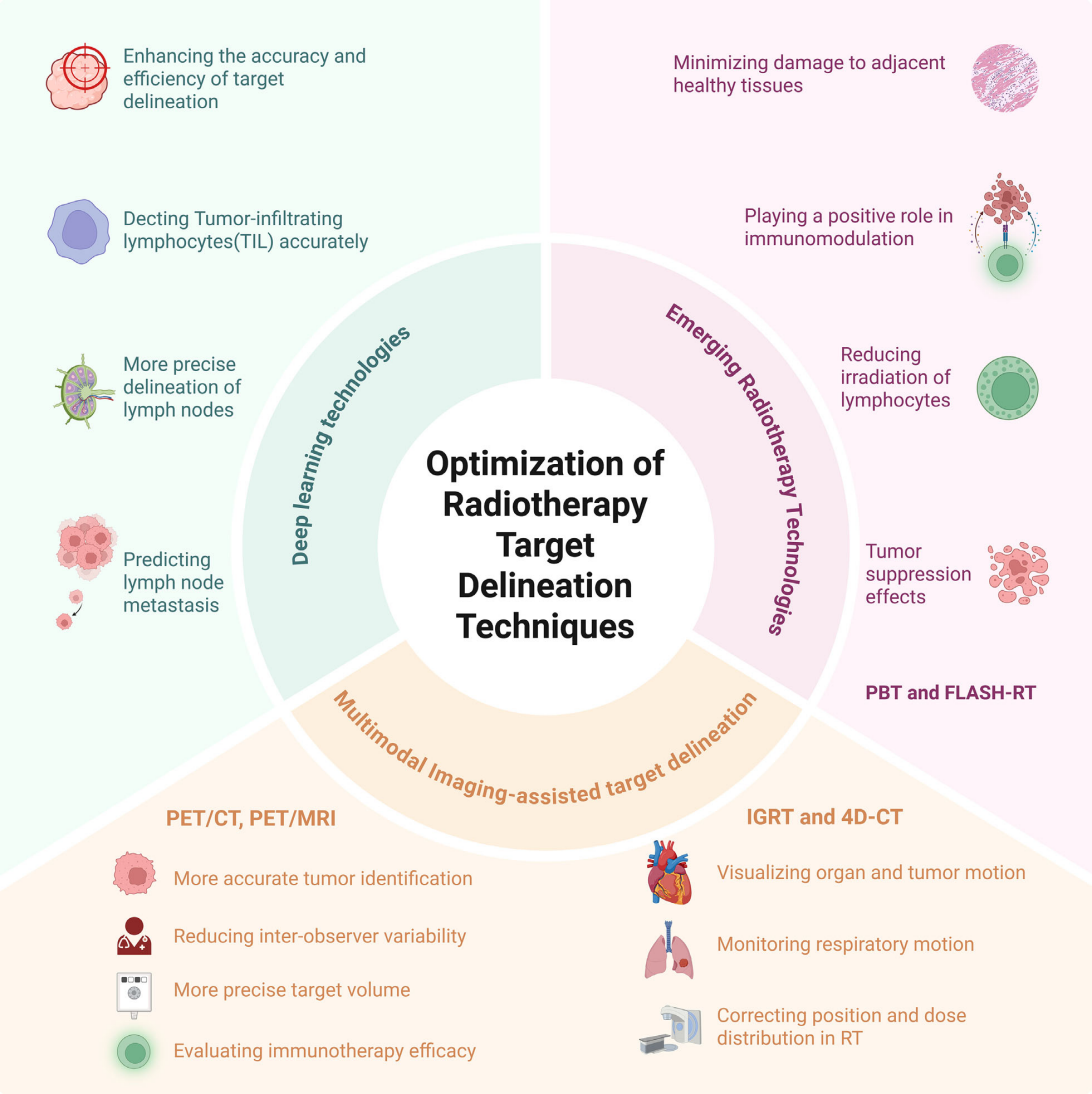
Brief summary of the optimization of radiotherapy target delineation techniques. Created in https://BioRender.com.

The immune‐protective effects of FLASH‐RT has opened doors for synergy with immunotherapy, potentially improving patient outcomes and quality of life. However, data regarding the combination of FLASH‐RT with immunotherapy remain extremely limited. The only preclinical study on this topic was conducted by Eggold et al.^131^ In 2022, FLASH increased T cell infiltration in patients with ovarian cancer and enhanced anti‐PD‐1 efficacy in comparison with CONV‐RT. Therefore, additional research is necessary to explore this area and enrich clinical evidence.(Figure 3)

## 5 DISCUSSION

Leveraging immunotherapy to amplify the immunostimulatory effects of RT while blocking its suppressive signals is important to optimize the effectiveness of the combination of RT and immunotherapy in thoracic tumors. To achieve this goal, new target delineation strategies—IFI, reduced irradiation volumes, CTV omission, and adjusted positioning—have shifted RT from broad anatomical coverage to minimal effective volume, preserving lymphatic integrity and supporting immunotherapy. Importantly, this paradigm shift does not simply pursue geometric volume reduction, but rather emphasizes functional optimization of the target coverage to balance tumor control with immune preservation. This strategy is supported by the aforementioned dose–volume effects observed in esophageal cancer, whereing sparing negative TDLNs was associated with improved systemic T cell activity and improving survival.^39^ Furthermore, emerging retrospective evidence has identified RIL as a key determinant of impaired immunotherapy efficacy, providing indirect mechanistic support for the rationale of target‐volume reduction. However, large‐scale phase III clinical trials directly comparing the efficacy of ENI versus IFI specifically in the context of PD‐1/PD‐L1 inhibitor therapy are currently lacking. Moreover, while the development of emerging technologies, such as multimodal imaging and real‐time dynamic tracking, have provided robust technical support for optimizing target delineation in thoracic tumors, these technologies still face challenges in practical applications. 4D imaging techniques are associated with issues such as long scanning times and variable image quality.^132^ Innovations such as sparse‐view reconstruction and intelligent scanning protocols are gradually addressing these challenges.^133^, ^134^, ^135^ Moreover, the existing multimodal imaging techniques are limited by a lack of functional biomarkers. Relying solely on traditional biomarkers such as ^18^F‐FDG cannot capture the full functional characteristics of tumors. In this regard, combining immunotherapy predictive biomarkers (like the tumor mutational burden^136^) with information obtained from anatomical imaging holds promise for enabling individualized target delineation on the basis of immune responses. Breakthroughs in spatial multiomics technologies also offer new perspectives: through 3D integration of transcriptomics, proteomics, and metabolomics, these approaches can map the spatial distribution and interactions of immune cells within the TIME,^137^ providing novel molecular foundations for precise target delineation.

Breakthroughs in artificial intelligence technologies have provided new momentum to precision RT, but the limitations associated with the use of DL include the complications in model training as a result of irregularities and boundary ambiguities of CTVs,^138^ difficulty in replicating subtle expert decisions in complex cases,^139^, ^140^ and the limited generalization to real‐world scenarios caused by shortcut learning.^141^ To address these, CNN‐based architectures like 3D U‐Net and Swin UNETR have demonstrated promising cross‐institutional robustness. Furthermore, emerging large‐scale models, including adaptations of the segment anything model (SAM), Mamba‐like architectures, and diffusion‐based approaches like Lung‐DDPM, have been explored for thoracic CT tasks. Although these approaches have shown potential for more robust and generalizable thoracic tumor segmentation,^142^, ^143^, ^144^ no mature studies have employed them directly to thoracic radiotherapy target delineation, making them a promising avenue for future research. Additionally, the use of digital twins (DTs) can yield virtual patient models to simulate treatment effects on the immune microenvironment, thereby facilitating a shift from the traditional one‐size‐fits‐all approaches to more targeted strategies.^145^, ^146^, ^147^ This approach has already shown advantages in proton therapy for other cancers, such as prostate cancer,^148^ and may be explored for thoracic tumors such as lung cancer in the future. The integration of DTs with artificial intelligence also shows promise in improving radiation dose prediction and optimizing treatment outcomes in the field of theranostics,^149^ marking a new era of “virtual‐real” interactions in RT and providing a dynamic optimization platform for immune‒protective target delineation.

Innovations in emerging RT technologies have opened new pathways for preserving immune function. Instead of fundamentally reducing target‐volume definitions, techniques such as proton therapy and FLASH‐RT primarily refine dose distribution within predefined targets, thereby limiting unnecessary irradiation of immune‐related normal tissues. However, proton therapy is associated with challenges such as uncertainties related to patient motion, setup, proton range, fluctuations in relative biological effectiveness (RBE), and insufficient clinical evidence.^108^, ^150^ Additional research is required to quantify the clinical benefits of proton therapy. FLASH‐RT, on the other hand, is limited by inadequate mechanistic studies, challenges in dosimetry and imaging technologies, and the need for specialized equipment.^151^, ^152^, ^153^, ^154^ Future advancements will require technological innovations and clinical validation to overcome these application bottlenecks. Several recent studies have explored proton FLASH‐RT for thoracic tumors, achieving encouraging results, which suggesting that proton FLASH therapy could be a promising alternative to traditional RT.^155^ Similarly, novel treatment approaches such as spatially fractionated radiotherapy (SFRT), which deliver high radiation doses to specific regions within the tumor, can effectively treat the entire tumor with heterogeneous dosing while maintaining the doses to surrounding healthy tissues^156^ within tolerable limits and simultaneously triggering robust immune responses.^157^ Trials such as NCT02303990 and NCT02383212 explored the use of SFRT with anti‐PD‐1 immunotherapy and reported stronger responses and abscopal effects.^158^ In conjunction with SFRT, heavy ion therapy (e.g., carbon ions) offers superior RBE and high linear energy transfer (LET), which can crucially overcome radioresistance in hypoxic thoracic tumors while further sparing peritumoral immune‐related structures.^159^, ^160^, ^161^ Future studies should aim to optimize the integration of these advanced modalities with immunotherapy to increase treatment precision and efficacy.

## CONFLICT OF INTEREST STATEMENT

The authors declare no conflicts of interest.

## ETHICAL STATEMENT

Not applicable.
